# Kinetics of SARS-CoV-2 infection biomarkers in a household transmission study

**DOI:** 10.1038/s41598-024-62835-0

**Published:** 2024-05-29

**Authors:** Ana M. Groh, Maria J. G. T. Vehreschild, Damian Diaz, Alison L. Kuchta, Christopher Dodoo, Luis A. Alvarado, Neil T. Parkin, Elissa M. Robbins, Priscilla Moonsamy, Tuna Toptan, Sandra Ciesek, Annemarie Berger

**Affiliations:** 1 Goethe University Frankfurt, University Hospital Frankfurt, Department 2 of Internal Medicine, Infectious Diseases, Frankfurt am Main, Germany; 2 Fraunhofer Institute for Translational Medicine and Pharmacology ITMP, Frankfurt am Main, Germany; 3 Roche Molecular Systems, Pleasanton, CA USA; 4 Data First Consulting, Inc., Sebastopol, CA USA; 5 Institute of Medical Virology, Goethe University Frankfurt, University Hospital Frankfurt, Frankfurt am Main, Germany; 6 Present Address: EP Statistical Consulting, LLC, El Paso, TX USA

**Keywords:** SARS-CoV-2, Viral transmission

## Abstract

SARS-CoV-2 is the causative agent of COVID-19. Timely and accurate diagnostic testing is vital to contain the spread of infection, reduce delays in treatment and care, and inform patient management. Optimal specimen type (e.g. nasal swabs or saliva), timing of sampling, viral marker assayed (RNA or antigen), and correlation with viral infectivity and COVID-19 symptoms severity remain incompletely defined. We conducted a field study to evaluate SARS-CoV-2 viral marker kinetics starting from very early times after infection. We measured RNA and antigen levels in nasal swabs and saliva, virus outgrowth in cell culture from nasal swabs, and antibody levels in blood in a cohort of 30 households. Nine household contacts (HHC) became infected with SARS-CoV-2 during the study. Viral RNA was detected in saliva specimens approximately 1–2 days before nasal swabs in six HHC. Detection of RNA was more sensitive than of antigen, but antigen detection was better correlated with culture positivity, a proxy for contagiousness. Anti-nucleocapsid antibodies peaked one to three weeks post-infection. Viral RNA and antigen levels were higher in specimens yielding replication competent virus in cell culture. This study provides important data that can inform how to optimally interpret SARS-CoV-2 diagnostic test results.

## Introduction

Severe acute respiratory syndrome coronavirus 2 (SARS-CoV-2) is the causative agent of coronavirus disease 2019 (COVID-19)^1–3^. The COVID-19 pandemic has led to widespread morbidity and mortality since first emerging in China in December 2019^4^. Timely and accurate diagnostic testing is vital to contain the spread of infection, reduce harmful delays in care delivery, and inform patient management. It is important to understand the performance of different assays throughout the course of an individual patient’s infection, including association of test results with infectiousness and disease severity and their ability to be used in combination to optimize patient management and inform public health action.

Detection and measurement of viral RNA using reverse transcriptase polymerase chain reaction (RT-PCR) is the gold standard for diagnosis of infection, because of its sensitivity and potential for rapid development and implementation^5^. Viral antigen testing is another useful tool for diagnosing acute infection, with less sensitivity compared to RT-PCR and other nucleic acid amplification tests, but good specificity, lower cost, and availability in multiple formats (e.g. home-, point of care, or laboratory-based rapid tests or laboratory-based automated high-throughput assays). Assays that detect the presence of circulating anti-SARS-CoV-2 antibodies are useful for identification of prior (resolved) infection, confirming response to vaccination, and monitoring population levels of immunity.

Multiple studies have attempted to describe SARS-CoV-2 viral kinetics and the relationship between viral antigen and RNA (RT-PCR) assays. In a meta-analysis of 79 studies, assessing 5340 individuals with SARS-CoV-2, the mean duration of SARS-CoV-2 RNA shedding was 17 days in the upper respiratory tract, with a peak in quantity in the first week of illness, and 14.6 days in the lower respiratory tract^6^. Some studies suggested that effective screening depends on frequency of testing and the speed of reporting, and is only marginally improved by high test sensitivity^7,8^. However, early detection of viral RNA by RT-PCR before antigen is detectable can be important in some situations, such as prevention of transmission to high risk patients in hospitals and care homes.

One clinically important aspect of diagnostic test results is their association with infectiousness (i.e., the degree to which an infected person is likely to infect others). An intuitive concept is that the ability to grow SARS-CoV-2 virus from a respiratory secretion specimen in cell culture is a proxy for the infectiousness of an infected individual^9^. In a 2021 meta-analysis, replication-competent virus was not detected beyond nine days from symptom onset, despite persistently high viral loads inferred from cycle threshold (Ct) values^6^. Another review reported a median last day of successful virus isolation of 11 days (95% CI 8.5–14.5 days), with longer times in critically ill patients^10^. The amount of time from symptom onset during which virus can be cultured in vitro from patient specimens is dependent on multiple variables, including the sensitivity of the virus culture method which in turn is influenced by the cell type used. Nonetheless it is clear that the predictive value of RNA detectability for infectiousness is low, due to the relatively high sensitivity of RT-PCR and the probable detection of viral RNA fragments from sources other than replication-competent virus. This discordance may be emphasized at later times from infection^11^. Interpretation of the clinical significance of RT-PCR results would benefit from definition of a threshold for quantitative measures of viral RNA in respiratory secretions^12,13^, but is complicated by the lack of standardization between methods. Detection of viral antigen may be more predictive for the ability to isolate replication-competent virus in vitro and of an individual’s infectiousness^14–18^.

A robust immune response to SARS-CoV-2 infection is typical in immunocompetent individuals, including humoral and cell-mediated responses to multiple viral antigens and epitopes^19^. Antibody levels and patterns (i.e. IgM vs IgG) have been associated with COVID-19 severity^20^. Measurement of anti-SARS-CoV-2 antibodies in blood is used to detect past infection after viral RNA and antigen are no longer present, for example in sero-prevalence and other epidemiological studies^21^, and to follow the response to vaccination.

Several different types of respiratory specimens can be used for diagnostic testing of SARS-CoV-2 infection including nasopharyngeal or oropharyngeal swabs (NPS or OPS), anterior nasal swabs, and saliva^22,23^. While NPS is considered the standard specimen type, anterior nares swabs and saliva are less invasive and amenable to self-collection, which can be advantageous for several reasons, such as the inconsistency and discomfort of collecting NPS when testing children or in adults when frequent testing is necessary.

We carried out a field study to evaluate SARS-CoV-2 viral biomarker kinetics starting from very early times after infection. We measured antigen and RNA levels in nasal swabs and saliva, virus outgrowth in cell culture from nasal swabs, and antibody levels in blood in a cohort of households in Germany. The objectives of this study were several-fold: (1) to describe viral and host response kinetics throughout SARS-CoV-2 infection as determined by antigen, antibody, and RNA testing in an early exposure population; (2) to evaluate each marker and their combinations as predictors of infectiousness (using virus culture positivity as a proxy); (3) to evaluate each marker and their combinations as predictors of disease severity, including hospital admission, healthcare visits and severe symptoms; and (4) to compare RNA and antigen test results obtained using saliva as a specimen type to those obtained from nasal swabs (the validated method).

## Results

### Study population and transmission

**Figure 1:**
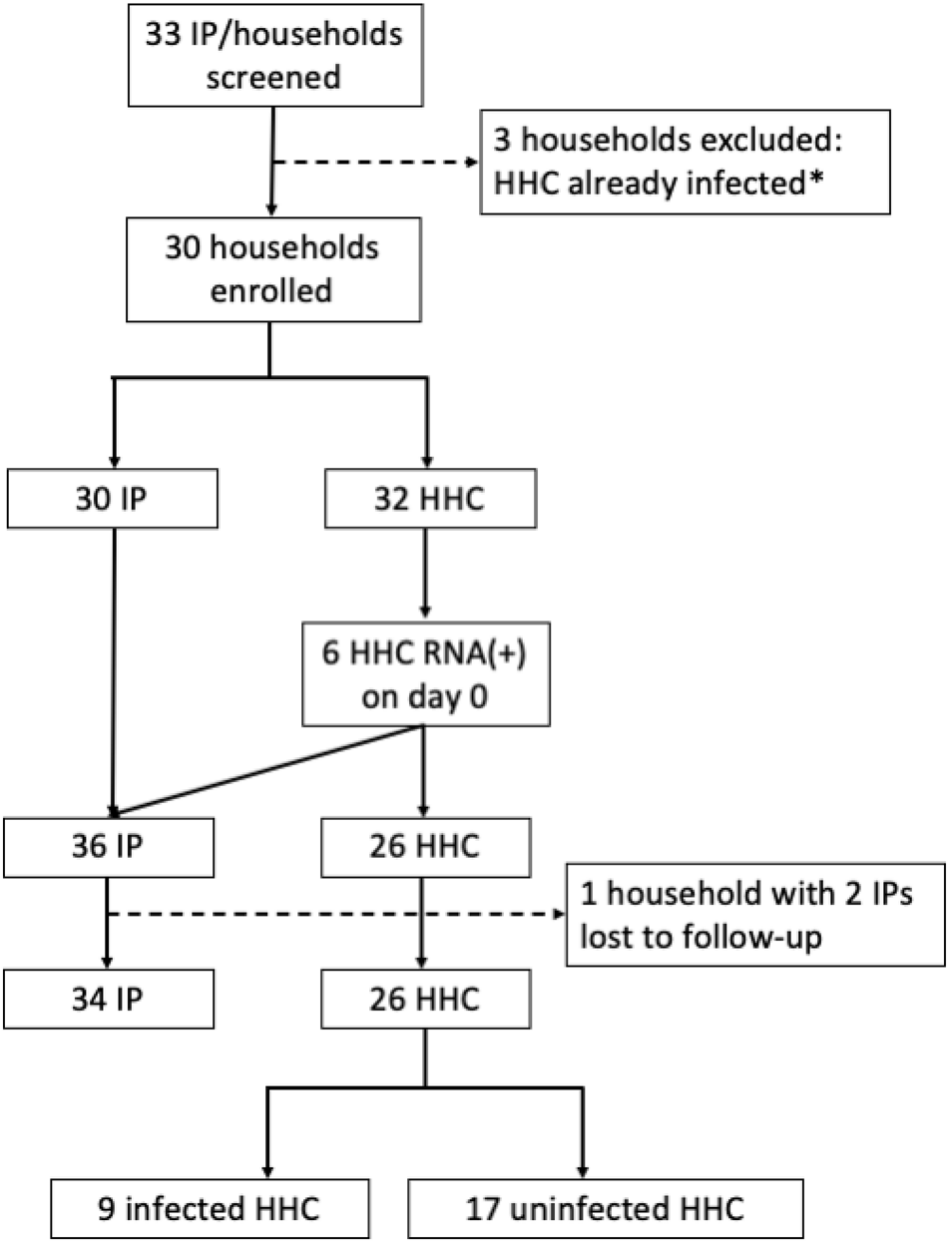
Study design and participant disposition. *Two households were excluded based on detection of SARS-CoV-2 RNA in nasal swabs from the HHC on day 0, one based on HHC symptoms consistent with COVID-19 in the 24 h prior to day 0. After these households were excluded, a protocol amendment was introduced to allow infected HHC to be reclassified as IP; this occurred in six households (diagonal arrow). IP, index patient; HHC, household contact; RNA(+), SARS-CoV-2 RNA detected in nasal swab specimen.

Study recruitment took place between June 2021 and October 2022. During this period, an active vaccination program was underway, and the predominant variant in Germany was Omicron. Thirty households were enrolled, each with one index patient (IP) and one household contact (HHC) except household number 9 which had three HHCs (Fig. 1). In six of the 30 households one HHC was found to be infected based on RNA testing in nasal swabs on day 0 and thus were reclassified as “exposed HHC” and treated as IP for analyses (households 7, 12, 15, 18, 25 and 28). After enrolment, one household (28) was lost to follow-up, leaving a total of 34 IP and 26 HHC in 24 households. Characteristics of these study participants are shown in Table 1.

**Table 1:** Participant numbers and basic characteristics.  IP, index patient; HHC, household contact.

	IP	HHC	Infected HHC	Uninfected HHC	Total
Number of subjects	34	26	9	17	60
Age, years—mean (range)	31 (19—57)	32 (20–55)	36 (23—55)	30 (20—53)	32 (19–57)
Sex (male) (N, %)	16 (47.1%)	10 (38.5%)	2 (22.2%)	8 (47.1%)	26 (43.3%)
Vaccinated (N, %)	33 (97.1%)	25 (96.2%)	9 (100%)	16 (94.1%)	58 (96.7%)
Type of vaccine (N, %)					
mRNA^a^	21 (61.8%)	19 (73.1%)	7 (77.8%)	12 (70.6%)	40 (66.7%)
Other or mix^b^	5 (14.7%)	6 (23.1%)	2 (22.2%)	4 (23.5%)	11 (18.3%)
None	1 (3.0%)	1 (3.9%)	0 (0%)	1 (5.9%)	2 (3.3%)
Missing data	7 (20.6%)	0 (0%)	0 (0%)	0 (0%)	7 (11.7%)
Symptoms (N, %)					
None	3 (8.8%)	7 (26.9%)	0 (0%)	7 (41.2%)	10 (16.7%)
1–2	1 (2.9%)	8 (30.8%)	2 (22.2%)	6 (35.3%)	9 (15.0%)
3 or more	30 (88.2%)	11 (42.3%)	7 (77.8%)	4 (23.5%)	41 (68.3%)

^a^Pfizer/BioNTech and/or Moderna.

^b^AstraZeneca, J&J in combination with each other or with Pfizer/BioNTech.

In nine of the 24 households, one HHC became infected (Table 2). The first positive RNA test using either assay or specimen type occurred in specimens collected between day 0 and 15. Five of the nine HHC were first positive between days 0 and 2, two on day 4, and two on day 15. All IPs in these households had at least one positive virus isolation in cell culture; in three households (9, 31 and 32) there was a delay of four days or more between the last positive culture from the IP and the first day that RNA was detected in the HHC. Nucleotide sequence data were available for the IP and HHC in households 10, 30, 31 and 32; phylogenetic analysis indicated that paired sequences in households 10 and 30 were more closely related to each other than the pairs from households 31 and 32 (Supplemental Material Fig. S1).

**Table 2:** Infected HHC summary.

Household	Day RNA first detected			Days from IP culture positivity^a^	Sequences from IP and HHC consistent with transmission	Comments
	cobas Liat, nasal swab	cobas 6800, nasal swab	cobas 6800, saliva			
4	4	4	4	0	na	
8	2	2	0	0	na	Saliva positive before nasal swab
9	21^b^	21^b^	15	15	na	Saliva positive before nasal swab; two other HHC in this HH not infected
10	4	1	0	0	Yes	Saliva positive before nasal swab; cobas 6800 positive before cobas Liat
17	2	2	0	0	na	Saliva positive before nasal swab
21	1	1	1	0	na	
30	2	2	2	2	Yes	
31	19	19	15	10	No	Saliva positive before nasal swab
32	6	6	4	4	No	Saliva positive before nasal swab

IP, index patient; HHC, household contact; na, sequence data not available.

^a^Days from closest prior/concurrent IP culture positive test and HHC 1st RNA detected.

^b^Previous scheduled visit on day 18 did not occur, day of RNA detectability in nasal swab may have been between days 16 and 21.

Because of the variable number of days between enrolment and transmission to HHC, for subsequent analysis we redefined the time when RNA first became detectable in nasal swabs in infected HHC as day 0.

### Clinical observations

As a group, the IPs and infected HHCs reported an average of five to six symptoms over the course of the study, while the uninfected HHC reported an average of fewer than two symptoms (Supplementary Material Fig. S2). IP and infected HHC also reported a higher frequency of moderate to severe symptoms, especially cough, sore throat, fatigue (Supplementary Material Fig. S3). No participant was hospitalized or died during the study. Additional analysis of participant symptom severity data is ongoing (manuscript in preparation).

### Dynamics of viral RNA and antigen and host antibody responses

**Figure 2:**
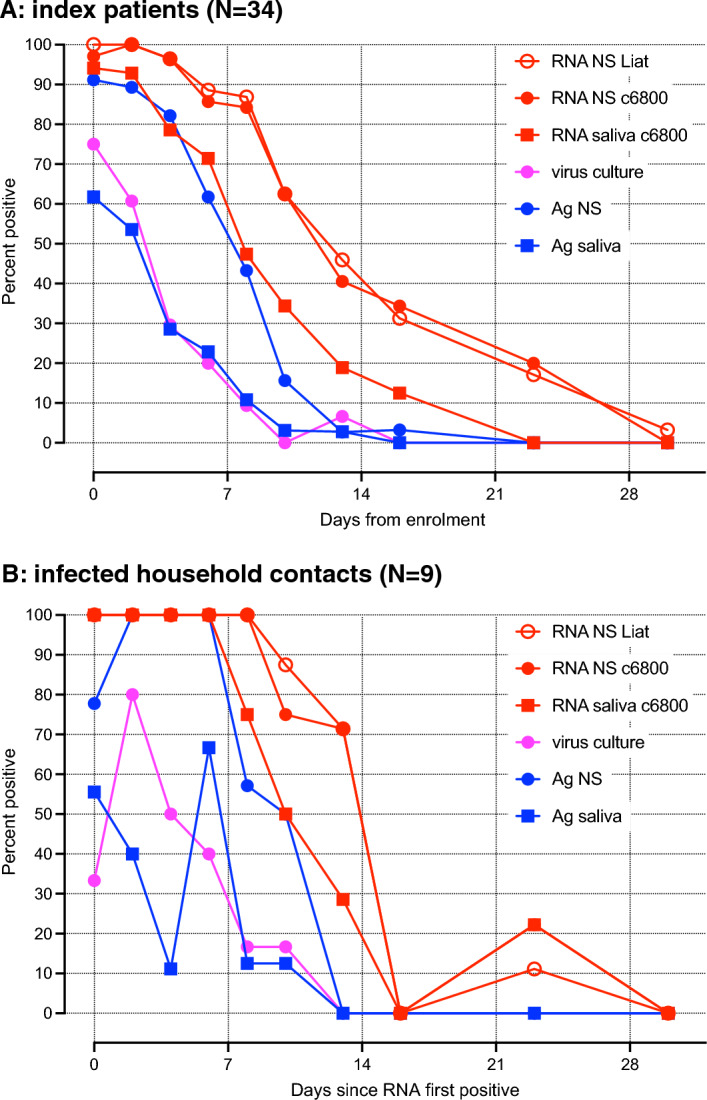
Percentage of participants with detectable viral RNA with the two different methods, antigen, or virus in cell culture in (**A**): index patients (IP); (**B**): infected household contacts (HHC). Nasal swab (NS) and saliva specimens were tested for RNA and antigen, and nasal swab specimens with RNA detected by cobas 6800 were tested for replication competent virus in cell culture. Days from enrolment (IP) or first positive RNA test (HHC) are grouped as described in “Methods”. Since virus culture was only attempted from nasal swab specimens with RNA detectable by cobas 6800, the proportion positive shown was adjusted by increasing the denominator by the number of negative RNA results (i.e. these were assumed to be virus culture negative).

SARS-CoV-2 RNA was assessed using two separate, real-time RT-PCR based tests. cobas® SARS-CoV-2 & Influenza A/B Nucleic acid test for use on the cobas Liat System (herein referred to as cobas Liat) and cobas SARS-CoV-2 for use on the cobas 6800/8800 Systems (herein referred to as cobas 6800; see “Methods”). The proportion of positive results for RNA in nasal swabs (using either cobas Liat or cobas 6800), RNA in saliva (cobas 6800), antigen in nasal swabs and saliva, and culturable virus in nasal swabs over time is shown in Fig. 2. In general, the detection of RNA by cobas Liat and cobas 6800 was comparable. RNA tests were negative in 28 of 35 (80%) specimens from IP at day 23, but remained detectable in one IP on day 30. In one household where the HHC became infected, the cobas 6800 result from nasal swab was positive on day 1 while the cobas Liat result was negative; both were positive on day 4. The detection of RNA (by cobas 6800) in saliva was less sensitive overall, compared to nasal swabs. However, in six of the nine HHC infections, RNA was detected in saliva one visit (one to six days) before it was positive in nasal swabs (Table 2). Antigen test positivity was lower than for RNA, as expected, and also lower in saliva compared to nasal swabs. Detection of antigen in nasal swabs was similar to that of RNA in saliva. Finally, the proportion of virus culture tests that were positive from nasal swab specimens was lower than for RNA or antigen. Virus culture was negative in 34 of 38 (89%) of specimens from day 8 (IP and HHC combined) and in all specimens collected more than two weeks from enrolment or the date of first detection of RNA.

**Figure 3:**
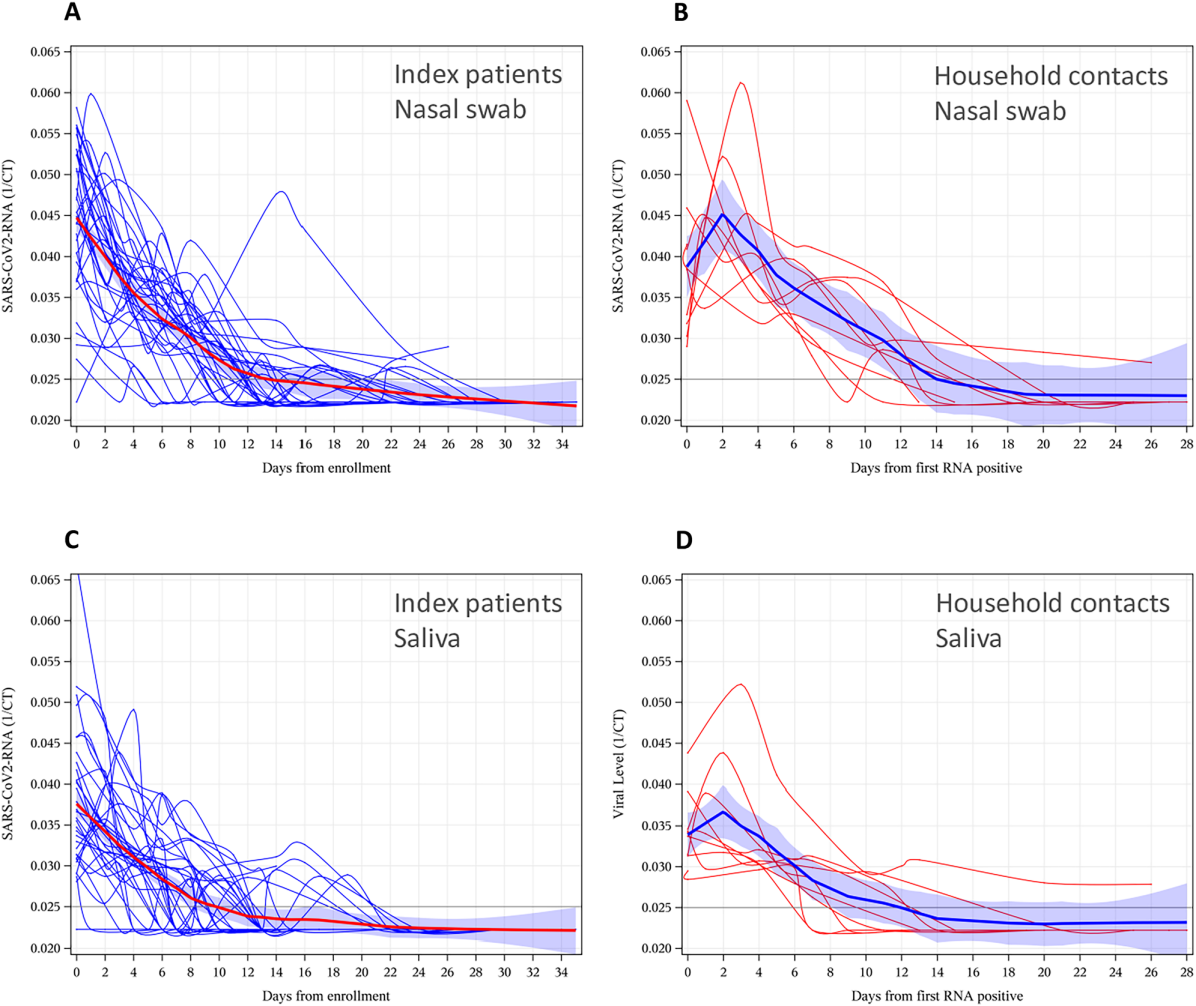
SARS-CoV-2 RNA levels represented by 1/Ct from the E gene target of the cobas 6800 assay. (**A**) and (**C**) (blue lines): index patients (IP; N = 34). (**B**) and (**D**) (red lines): infected household contacts (HHC; N = 9). (**A**) and (**B**): nasal swabs; (**C**) and (**D**): saliva. X-axis is days from enrolment (day 0) for IP and days from first positive RNA test for infected HHC. The spline curve (locally weighted polynomial regression with a smoothing parameter of 0.3) in each plot is shown in red (IP) or blue (infected HHC) with 95% confidence band shaded in blue.

The relative quantity of RNA in nasal swabs, as indicated by the reciprocal of the Ct number (1/Ct) for target 2 (E gene), is shown in Fig. 3 for IP (Fig. 3A) and infected HHC (Fig. 3B). Results for individual participants in households where the HHC became infected are shown separately in Supplementary Material Fig. S4. RNA levels decreased sharply from enrolment in 26 participants, while in 14 the peak RNA level was reached one to three days later. In one striking case (HH 26), RNA levels decreased from enrolment to day 6, then increased again reaching a peak on day 14; it was undetectable by day 30. Virus culture testing was positive for this participant on day 0 and 14. This unusual pattern is likely related to treatment with nirmatrelvir/ritonavir followed by recrudescence of viral replication due to incomplete viral clearance^24^.

The pattern of RNA levels over time in saliva specimens was similar to that of nasal swabs, although RNA in saliva became undetectable in IPs sooner than for RNA in nasal swabs (Fig. 3C and D). Interestingly the increase observed on day 14 in IP 26 was not observed in saliva.

Virus isolation in cell culture was attempted using nasal swab specimens in which viral RNA was detectable with the cobas 6800 assay. Of 248 specimens, 74 (29.8%) were positive. In IPs, the percent culture positive was highest on day 0 (24 of 32, 75%), decreasing on day 2 (17 of 30, 57%) and thereafter (0 to 32%). In infected HHC, the percent culture positive was highest on day 2 (4 of 5, 80%), decreasing on day 4 (4 of 8, 50%) and thereafter (0 to 36%).

**Figure 4:**
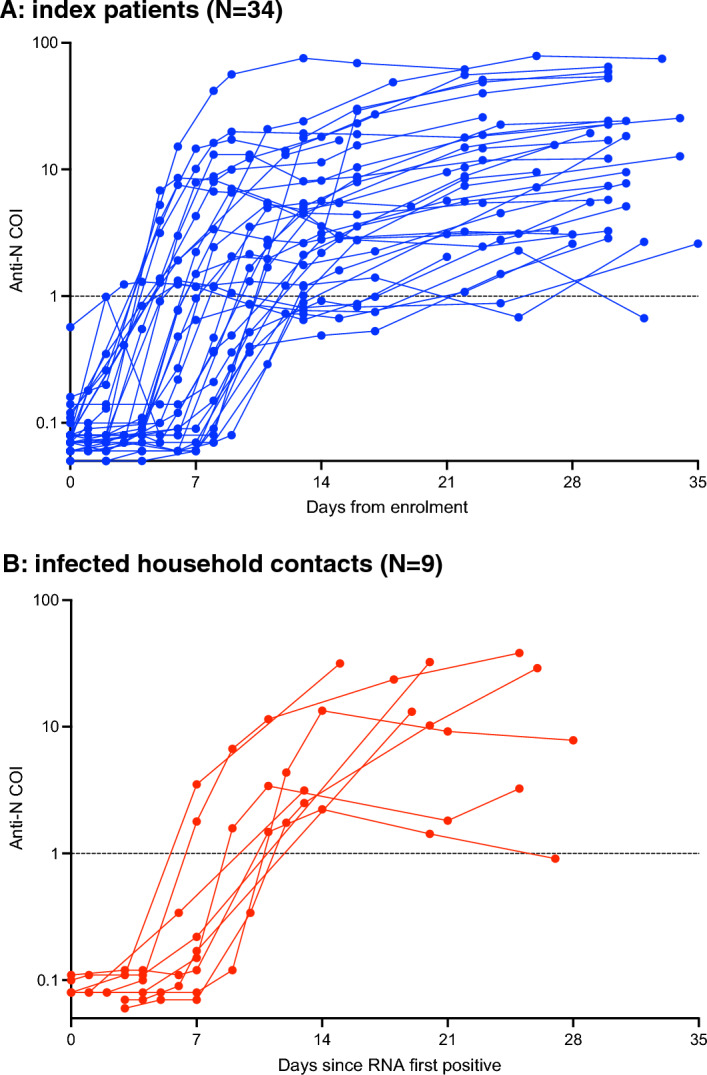
Anti-N antibody cut-off index (COI) for each individual index patient (**B**, blue) and infected household contact (**C**, red). Horizontal dotted lines indicate the cutoff for positivity.

Antibodies to the receptor-binding domain of SARS-CoV-2 spike protein (anti-S) were detectable at day 0 in all IP and infected HHC, except for one unvaccinated IP. By day 4 and thereafter, all IP and infected HHC had detectable anti-S antibodies (Fig. 4). In contrast, antibodies to the nucleocapsid protein (anti-N) were not detected in any participant until day 6 from enrollment (IP) or day 8 from first RNA positive test (infected HHC). Anti-N antibodies were detected in over 80% of IP and all HHC from day 13 onward (Supplemental Material Fig. S5). Anti-N antibody titers increased with variable kinetics in individual participants; in some cases, titers were close to the threshold for positivity and fell just above or below the threshold for reactivity although the signal intensity was above the baseline level. Anti-S antibody concentrations increased roughly three-fold from day 0 to 16 (data not shown). All 418 anti-S reactive specimens are also reactive with the SARS‑CoV‑2 Rapid Antibody Test (data not shown).

### Assay comparisons

We compared the performance of the different tests and specimen types with respect to their ability to accurately diagnose SARS-CoV-2 infection. Detection of viral RNA in nasal swabs by RT-PCR was considered the gold standard against which we compared detection of viral RNA in saliva by RT-PCR, and viral antigen in nasal swabs. The positive percent agreement (PPA) for RNA in saliva was 71% compared to nasal swabs, while the negative percent agreement (NPA) was 95% (Table 3). Six of the 17 specimens that were positive in saliva but negative in nasal swabs were from HHC on the first day of RNA positivity; eleven were from IP, of which only one was on day 0, while ten were from day 5 or later.

**Table 3:** RNA detectability in nasal swab vs saliva (cobas 6800). PPA, positive percent agreement; NPA, negative percent agreement; OPA, overall percent agreement; CI, confidence interval.

		Nasal swab		Total
		N RNA +	N RNA −	
Saliva	N RNA +	177	17	194
	N RNA −	73	311	384
	Total	250	328	578
PPA: 70.8% (95% CI: 64.9, 76.1) NPA: 94.8% (95% CI: 91.9, 96.7) OPA: 84.4% (95% CI: 81.2, 87.2)				

Antigen tests are known to be most sensitive in individuals with symptoms of COVID-19 at early times after infection^25–27^. Therefore we limited our analysis of antigen test performance to specimens collected on day 6 or earlier from symptomatic study participants. The PPA of antigen testing in nasal swabs compared to RNA by cobas Liat or cobas 6800 was between 85 and 86% (Table 4), while the NPA was 98 to 100%. There was a single specimen that was negative for RNA by cobas Liat that was antigen reactive from an infected IP on day 0. Amongst the 17 specimens with RNA detectable by cobas 6800 but non-reactive antigen test results, the E gene Ct values ranged from 28.5 to 37.6; 16 of 17 results were over 30; the mean Ct value was 33.9. The PPA was lower if all samples were included (Supplemental Material Table S1).

**Table 4:** RNA vs Ag detectability in nasal swabs (first 6 days, symptomatic patients). PPA, positive percent agreement; NPA, negative percent agreement; OPA, overall percent agreement; CI, confidence interval.

		RNA (cobas Liat)			RNA (cobas 6800)		
		N Positive	N Negative	Total	N Positive	N Negative	Total
Antigen	N Reactive	104	1	105	105	0	105
	N Non-reactive	19	57	76	17	59	76
	Total	123	58	181	122	59	181
	PPA: 84.6% (95% CI: 77.1, 89.9) NPA: 98.3% (95% CI: 90.9, 99.7) OPA: 89.0% (95% CI: 83.5, 92.7)				PPA: 86.1% (95% CI: 78.8, 91.1) NPA: 100% (95% CI: 93.9, 100.0) OPA: 90.6% (95% CI: 85.5, 94.1)		

**Figure 5:**
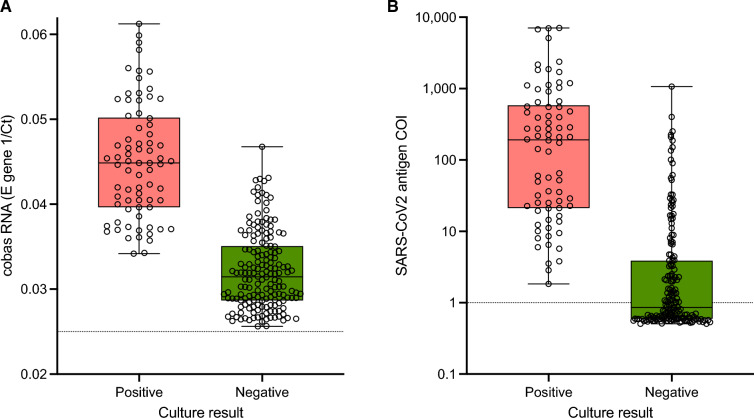
(**A**) RNA levels represented by 1/Ct from the E gene target of the cobas 6800 assay and (**B**) antigen levels (signal intensity) in specimens according to viral culture positivity (positive, salmon; negative, green). Horizontal bars in the boxes represent the median, the box boundaries represent the inter-quartile range, and whiskers represent the range. Horizontal dotted lines indicate the cutoff for positivity. Note that specimens with undetectable RNA were not tested in cell culture. COI, cut-off index.

The presence of infectious SARS-CoV-2 in nasal swab specimens with detectable RNA was assessed by inoculation of Caco-2 cells in culture. The relative quantity of RNA in specimens that were culture positive was significantly higher than in specimens that were culture negative (median Ct value 31.8 vs. 22.3; Fig. 5A). There was considerable overlap in Ct values amongst culture positive and negative specimens; in specimens with Ct values between 21 and 30 (or 1/Ct between 0.033 and 0.048), 48.1% were culture positive. No samples with Ct values above 30 (or 1/Ct below 0.033) were culture positive. Amongst specimens with Ct of 30 or lower, 56.9% were culture positive; the percentage increased to 80.5% for Ct 25 or lower. In symptomatic patients at day 6 or earlier, 46.0% of the 124 specimens with RNA detectable by cobas Liat and 46.3% of the 123 specimens with RNA detectable by cobas 6800 yielded positive virus cultures.

Similarly, the relative quantity of viral antigen (reflected by the assay cut-off index value, COI) in specimens that were culture positive was significantly higher than in specimens that were culture negative (median COI 191 vs. 0.86; Fig. 5B). Again, there was considerable overlap in signal intensity amongst culture positive and negative specimens. No samples with COI values below 1.8 were culture positive.

## Discussion

In this study of SARS-CoV-2 household transmission, nine instances of new infection were directly observed beginning from the first day on which RNA could be detected. We were also able to follow 34 infected IP starting from the initial few days of infection. This allowed us to follow the changes in viral RNA, antigen, virus infectivity, and host immune response over time.

In six of the nine new infections, viral RNA became detected in saliva specimens before doing so in nasal swabs (Table 2). Saliva and nasal swab samples are from different anatomical sites and differences may be expected, however, this observation is in contrast to an overall higher sensitivity for detection of viral RNA in nasal swabs compared to saliva (Table 3). A greater proportion of saliva positive, nasal swab negative results were derived from very early times after infection compared to nasal swab positive, saliva negative ones. These results suggest that at very early times after virus transmission, sampling of saliva may be advantageous with regard to early detection of infection. Our results are consistent with those reported by others using different RNA detection methods and showing that viral loads were higher in saliva or that viral RNA was detected in a higher proportion of saliva vs nasal swab specimens^28–31^. More efficient detection in saliva may be in part due to less invasive and more consistent specimen collection compared with swab-based methods, particularly in children^32^. Other factors including whether the specimen (saliva or swab) is collected by a trained care provider or self-collected, whether the donor gargles, clears their throat, drinks, or eats before producing the specimen, are likely to influence the efficiency of RNA detection in saliva, which may explain why some reports have not reproduced this finding. Earlier detection of infection, even by a day, can accelerate initiation of antiviral treatment and mitigation of onward transmission through isolation.

No single laboratory test can accurately predict whether an individual infected with SARS-CoV-2 is likely to be infectious to others. Intuitively, the ability to grow virus in cultured cells, an indication of the presence of replication-competent virus, from a respiratory secretion specimen implies that the individual from whom it was sampled was at least potentially infectious at the time of sampling^33,34^. However, there are many reasons why the converse (inability to grow virus in culture implying non-infectiousness) may not be true, such as the presence of substances in the specimen that interfere with cultured cell viability or of the ability of virus to infect the cells (e.g. anti-SARS-CoV-2 antibodies^35^), as well as the relative quantity of the angiotensin converting enzyme-2 receptor, cellular proteases required for virus entry (e.g. TMPRSS-2^36,37^), or other factors that impact the permissiveness of the cultured cells used. This can lead to discordance between the minimum virus concentration required for person-to-person transmission and that required for productive infection of cells in vitro. It is well established that qualitative detection of viral RNA is poorly predictive of cell culture infectivity, especially in specimens collected late in the course of infection. Detection of viral antigen is less sensitive than of viral RNA using RT-PCR, and has higher predictive value^38–40^. The results from our study are consistent with these observations, and help provide the context in which individual RNA or antigen test results are interpreted. Based on our data that show greater concordance between detection of antigen in either specimen type or of RNA in saliva compared to that of RNA in nasal swabs (Fig. 2), it is likely that detection of these analytes (and especially of antigen in saliva at early times after infection) is more predictive of infectiousness than that of RNA in nasal swabs. Ideally, analyte concentrations would be measured reliably in each specimen type and related to minimum infectious dose in clinical studies. Reproducible measurement of viral RNA with RT-PCR assays requires wide assay linearity and calibration using standardized reference control material and cannot be inferred directly from Ct values.

Almost all participants included in this study were vaccinated with S-encoding mRNA or adenovirus vector vaccines prior to enrolment; anti-S antibodies were already present in these participants before infection. It is not clear whether infection still occurred because the concentration of circulating or mucosal anti-S antibodies was not high enough, or because the infecting strain of SARS-CoV-2 was not sufficiently well recognized by these antibodies. In contrast, the host response to infection was clearly evident in the development of anti-N antibodies in all infected participants within about 2 weeks of infection, albeit reaching different maximal levels in each individual.

Our study has several limitations. As suggested by the sequence analysis and by the delay between IP infectiousness and HHC first RNA positive date, it is likely that at least some of the HHC were infected by someone other than the IP in their household. In addition to the relatively small sample size, this limits our ability to make conclusions about within-household factors or individual IP or HHC behaviors and characteristics that might be associated with transmission. Since the variants of SARS-CoV-2 that were circulating during the study are different from those circulating now or in the future, our observations may not be broadly generalizable. The antigen testing was performed in a central laboratory, and so our observations regarding antigen testing are not likely to apply to rapid (self) testing in all situations. It was not possible to directly compare the quantity of virus in saliva to that in nasal swab specimens, since the sampling method and volume of diluent used was different. Virus culture was only attempted in specimens with detectable RNA, making it challenging to assess the concordance of different analyte detection with culture positivity; in addition, freezing and storage of a small number of specimens may have affected the ability to recover viable virus in cell culture. Finally, the assignment of day 0 in HHC as the first day of detection of RNA in nasal swabs may not result in perfect alignment of the time course of infection compared to the IPs, since they were enrolled following one earlier positive RNA test.

In conclusion, these data add to an existing body of knowledge about the dynamics of viral and host biomarkers in the early stages of infection by SARS-CoV-2. Our observations suggest a potential role for saliva as a specimen type for early detection of infection and provide contextual information that can guide clinical interpretation of RNA, antigen and virus culture test results.

## Methods

### Study design

This was a natural history study evaluating several diagnostic tests detecting biomarkers related to COVID-19 conducted between June 2021 and October 2022. Ethical approvals were obtained from Ethikkommission des Fachbereichs Medizin der Goethe-Universität c/o Universitätsklinikum (Reference number: 2021-119-MPG) and all procedures were performed in accordance with the relevant guidelines and regulations. Two groups of subjects were enrolled: SARS-CoV-2-infected IP and their HHC. Individuals who were identified to be infected with SARS-CoV-2 at the acute care clinic at University Hospital Frankfurt, based on RT-PCR testing in nasal swab specimens and meeting IP eligibility criteria (see Supplemental Methods), were recruited. While the protocol allowed for the inclusion of anyone over the age of 15 years, no participants under the age of 19 were enrolled. Informed consent was obtained after verification of eligibility. IP were not required to be symptomatic for enrollment. After an IP was enrolled, a healthcare professional contacted the HHC(s) to explain the study commitment, assess eligibility, and obtain informed consent. The SARS-CoV-2 status of each IP and HHC was evaluated on visit 1 (day 0) using two RT-PCR tests (see below) in nasal swab specimens. These results as well as other criteria including not having more than one symptom commonly related to COVID-19 (for HHC; see Supplemental Methods) determined whether the subjects were eligible for the study. If an HHC had a positive nasal swab RNA test on day 0, they were classified as “exposed HHC” and considered as an IP in the analysis.

### Specimen collection

All specimens were collected by healthcare professionals at the participant’s household and held in a cooler with ice packs. Specimens were transported on the same day in a cooler with ice packs to the Institute of Medical Virology, University Hospital Frankfurt laboratory where they were stored at 2–8 °C until tested or frozen at – 20 °C within 48 h. Anterior nasal swab specimens were collected using flocked swabs (part number 306C, Copan, Murrieta, CA) in 3 mL universal transport medium (Copan). Remnant nasal swab specimens were frozen at − 80 °C.

Before saliva specimen collection, participants were asked to refrain from eating, drinking, smoking/vaping, using mouthwash or chewing gum for 30 min. Saliva accumulation in the mouth was encouraged by rubbing the inside of the cheek with the tongue or gently massaging the outside of the cheek. Saliva specimens (> 1 mL) were collected into 50 ml conical tubes (avoiding coughing or clearing the throat). At the laboratory, 1 mL of the saliva was added to 2 mL of phosphate buffered saline and mixed by vortexing. Remnant saliva specimens were frozen at − 80 °C.

Blood specimens were drawn by venipuncture into standard 10 mL serum collection tubes. Serum was separated by centrifugation in the laboratory and stored at − 80 °C.

### Laboratory tests

All products used in this study had received local regulatory clearance for identifying the corresponding biomarkers according to their intended uses in at least one specimen type. Off-label (e.g. detection of SARS-CoV-2 RNA or antigen in saliva specimens) results were not shared with the study subjects and were not used for treatment decisions. Testing of unfrozen specimens at the University Hospital Frankfurt laboratory was performed within 48 h of collection.

SARS-CoV-2 RNA was assessed using two separate, real-time RT-PCR based tests. cobas Liat is an automated multiplex real-time RT-PCR assay intended for the simultaneous rapid in vitro qualitative detection and differentiation of SARS-CoV-2, influenza A, and influenza B virus RNA^41^. The cobas Liat assay relies on amplification of two different targets in the SARS-CoV-2 genome (located in the ORF1a/b and N genes) and reports qualitative (positive/negative) results^42^. cobas 6800 is a real-time RT-PCR test intended for the qualitative detection of nucleic acids from SARS-CoV-2. The cobas 6800 assay also relies on amplification of two different targets in the SARS-CoV-2 genome (located in the ORF-1a/b and E genes) that are reported individually^43^. Results are reported as positive (if either target is detected) or negative. Ct values from both the cobas Liat and cobas 6800 assay are inversely related to the relative concentration of viral RNA in the specimen but have not been calibrated or standardized to a specific RNA copy number or other units. Here we used the reciprocal of Ct (i.e. 1/Ct) for the cobas 6800 E gene target to represent relative viral RNA quantity^43^. All RNA testing was performed in the University Hospital Frankfurt laboratory.

SARS‑CoV‑2 nucleocapsid antigen was assessed using the qualitative Elecsys® SARS‑CoV‑2 Antigen electrochemiluminescence (ELICA) immunoassay^44^ on a cobas e immunoassay analyzer at Roche in Penzberg, Germany. The Elecsys SARS‑CoV‑2 Antigen assay demonstrated high specificity (99.9%) and a sensitivity of 93.7% for samples containing more than 10^4^ viral RNA copies/mL^45^.

Anti-N antibodies were assessed using Elecsys Anti‑SARS‑CoV‑2 for qualitative detection of antibodies. The Elecsys Anti-SARS-CoV-2 immunoassay was performed at Roche in Penzberg, Germany, according to the manufacturer’s instructions. Assay results were interpreted as nonreactive/negative if the cutoff index was below 1.0 and reactive/positive if the cutoff index was 1.0 or greater^46,47^.

Anti-S antibody titers were measured using the Elecsys Anti‑SARS‑CoV‑2 S immunoassay, performed at Roche in Penzberg, Germany, according to the manufacturer’s instructions. Results are automatically calculated based on a lot-specific standard curve and reported as the analyte concentration of each sample in units per mL^48–50^. Assay results were interpreted as nonreactive/negative for concentrations below 0.8 U/mL and reactive/positive for concentrations equal to or greater than 0.8 U/mL.

We also used the SARS‑CoV‑2 Rapid Antibody Test, a rapid chromatographic immunoassay intended for the qualitative in vitro detection of antibodies to SARS‑CoV‑2 in human serum, plasma or whole blood. The rapid antibody test detects IgG and IgM antibodies against S and N combined^51,52^ and was performed at the University Hospital Frankfurt laboratory.

Virus outgrowth in cell culture was attempted with specimens in which RNA was detectable. In most cases, virus culture testing was initiated within 24 h of specimen collection. If this was not possible, specimens were frozen at – 20 °C until testing. Virus isolation was performed as previously described^53^. Briefly, swabs were mixed with minimal essential medium containing 1% fetal calf serum (Sigma-Aldrich; St. Louis, MO, USA), 3% amphotericin B, and 0.2% Primocin (InvivoGen; San Diego, CA, USA). 300 µL of the mixture was immediately transferred to Caco-2 cells (human colon carcinoma cells; DSMZ, Braunschweig, Germany, No.: ACC 169), and seeded in 5.5 cm^2^ culture tubes. Cells were incubated in a CO_2_ incubator at 37 °C for up to 7 days and assessed microscopically every day for virus specific cytopathogenic effects.

Sequencing of SARS-CoV-2 from selected nasal swab specimens with high RNA levels was performed using Nanopore sequencing with Artic V4.1 primers and bioinformatic analyses as previously described^54^.

### Visit schedules

Specimens were collected from IPs and HHCs in each household according to a flexible schedule that was adjusted when a HHC became infected. The schedule of visits and testing plan are illustrated in Supplemental Material Fig. S6.

As long as the HHC(s) remained uninfected, visits took place at approximately 2-day intervals during the first 10 days (visits 1–6), then 3-day intervals for the next two visits (7 and 8), then weekly for 2 more weeks (visits 9 and 10) for a total of 30 days of follow-up (Fig. S6A). In practice, the actual timing of each visit had a window of ± 1 day for the first five visits, ± 2 days for visits 6–8, and ± 3 days thereafter. Nasal swab, saliva and blood specimens were collected from IPs at every visit. If the HHC remained uninfected, nasal swab and saliva specimens were collected from HHC through visit 8 (day 16), while blood was collected for antibody testing only at visits 1 and 10.

If the HHC became infected (as indicated by a positive RNA test in the nasal swab specimen) within the first week (i.e. by visit 4) of observation, blood specimens were collected starting at the next visit and at each visit thereafter through visit 10 (day 30). If the HHC became infected on visit 5 or later, blood specimens were collected starting at the next visit, and the frequency of visits thereafter was increased for the next week as shown in Fig. S6A; duration of follow up was increased to a total of approximately 30 days from the date on which the HHC first tested positive.

Nasal swab specimens were tested for RNA using both cobas 6800 and cobas Liat, and for antigen (Elecsys). Virus culture was attempted if RNA was detectable by cobas 6800 in nasal swab specimens collected within seven visits of the first RNA positive specimen. Saliva specimens were tested for RNA using cobas 6800, and for antigen using Elecsys SARS‑CoV‑2 Antigen immunoassay. Serum specimens were tested for anti-SARS-CoV-2 antibodies using Elecsys Anti‑SARS‑CoV‑2, Elecsys Anti‑SARS‑CoV‑2 S, and the SARS‑CoV‑2 Rapid Antibody Test.

Because HHC became infected after a variable amount of time from IP enrolment, for illustrative purposes we defined day 0 for infected HHC as the day or first positive RNA test by cobas Liat or cobas 6800. The proportion of participants testing positive for different analytes over time was plotted using a categorical grouping of actual days from enrolment (IP) or first positive RNA test (HHC) as follows: day 2 (actual days 1–2), day 4 (days 3–4), day 6 (days 5–6), day 8 (days 7–8), day 10 (days 9–11), day 13 (days 12–14), day 16 (days 15–19), day 23 (days 20–26) and day 30 (day 27–40).

### Statistical analysis

We employed various descriptive statistics to summarize the continuous level data, such as the mean, median, standard deviation, and range. For categorical data, sample proportions were utilized. We calculated two-sided 95% Wilson score confidence intervals for concordance analyses (i.e. PPA, NPA, and overall percent agreement: OPA). To assess the significance of our findings, we set an alpha level of 0.05 for hypothesis testing. Any p-values below this threshold were considered statistically significant. All statistical analyses were performed using SAS v9.4 (The SAS Institute, Cary, NC, USA).

### Supplementary Information

Supplementary Information.

## Data Availability

The datasets generated during and/or analyzed during the current study are not publicly available due to patient confidentiality but are available from the corresponding author on reasonable request.
