# Supplementary material for: Kinetics of SARS-CoV-2 infection biomarkers in a household transmission study
Source: Sci Rep. 2024 May 29;14:12365. doi: 10.1038/s41598-024-62835-0 (PMC11136983; doi:10.1038/s41598-024-62835-0)
Supplement: Supplementary file 1 — Supplementary Information. [file 41598_2024_62835_MOESM1_ESM.docx]

**Kinetics of SARS-CoV-2 Infection Biomarkers in a Household Transmission Study**

**Groh et al.**

**Supplemental Material**

**Contents**

[Supplemental Methods 2](#_Toc156220535)

[Supplemental Results 3](#_Toc156220536)

[Table S1. RNA vs Ag detectability in nasal swabs (all participants, all visits) 3](#_Toc156220537)

[Figure S1. SARS-CoV-2 sequence relatedness. 4](#_Toc156220538)

[Figure S2. Number of symptoms reported. 5](#_Toc156220539)

[Figure S3. Symptom severity according to patient type. 6](#_Toc156220540)

[Figure S4. Kinetics of SARS-CoV-2 RNA levels. 7](#_Toc156220541)

[Figure S5. Antibody responses. 8](#_Toc156220542)

[Figure S6. Study design and testing strategy. 9](#_Toc156220543)

# Supplemental Methods

- Index Patients (IP) Inclusion Criteria
  - 15 years of age or older
  - At least one eligible household contact (HHC; according to criteria listed below at the time of consent)
  - Confirmed diagnosis of SARS-CoV-2 by RT-PCR or antigen test at the time of consent
  - Provided informed consent.
- Index Patients Exclusion Criteria
  - Women who are or become pregnant anytime during the study or who are breastfeeding.
  - Subsequent negative SARS-CoV-2 result by both Roche PCR tests from specimens collected from At-Home Visit 1 (Day 0), unless an HHC in this household tested positive, in which case, that HHC with SARS‑CoV-2 will become the new IP and the original IP will be part of the HHC.
- Household Contacts Inclusion Criteria
  - An HHC was defined as any person who had resided in the same household (or other closed setting) as the confirmed COVID-19 IP. A household was defined as a group of two or more people living in the same residence. In practice, the technical definition varied due to social, political and cultural practices. Definitions of a household that may have been used included but were not limited to the following: a) two or more people living together in a domestic residence (residential institutions, such as hostels, or prisons will be excluded); and b) a dwelling with a shared kitchen or common opening onto a shared household space.
  - 15 years of age or older
  - Has not been previously diagnosed with COVID-19 at the time of consent.
  - Has not experienced 2 of more symptoms commonly related to COVID-19, as listed below, within the past 24 hours of consent.
    - Fever
    - Cough
    - Headaches
    - Fatigue
    - Muscle or body aches
    - Loss of taste or smell
    - Sore throat
    - Nausea
    - Diarrhea
  - Provided informed consent.
- Household Contacts Exclusion Criteria
  - Women who are or become pregnant anytime during the study.
  - Women who are breastfeeding

# Supplemental Results

## Table S1. RNA vs Ag detectability in nasal swabs (all participants, all visits)

|  |  | **RNA (Liat)** | | |  | **RNA (c6800)** | | |
| --- | --- | --- | --- | --- | --- | --- | --- | --- |
|  |  | **Positive** | **Negative** | **Total** |  | **Positive** | **Negative** | **Total** |
| **Antigen** | **Reactive** | 154 | 1 | 155 |  | 155 | 0 | 155 |
|  | **Non-reactive** | 96 | 322 | 418 |  | 92 | 326 | 418 |
|  | **Total** | 250 | 323 | 573 |  | 247 | 326 | 573 |
|  | PPA: 61.6% (55.4, 67.4)  NPA: 99.7% (98.3, 99.9)  OPA: 83.1% (79.8, 85.9) | | | |  | PPA: 62.9% (56.6, 68.5)  NPA: 100% (98.8, 100.0)  OPA: 83.9% (80.7, 86.7) | | |

## Figure S1. SARS-CoV-2 sequence relatedness.

A neighbor-joining tree generated using available genome sequences aligned with Geneious Prime 2022 is shown. Labels are IP (index patient) or HHC (infected household contact) with the days from IP enrolment (unadjusted). Pairs of sequences from IP and HHC in the same household are color coded.

## Figure S2. Number of symptoms reported.


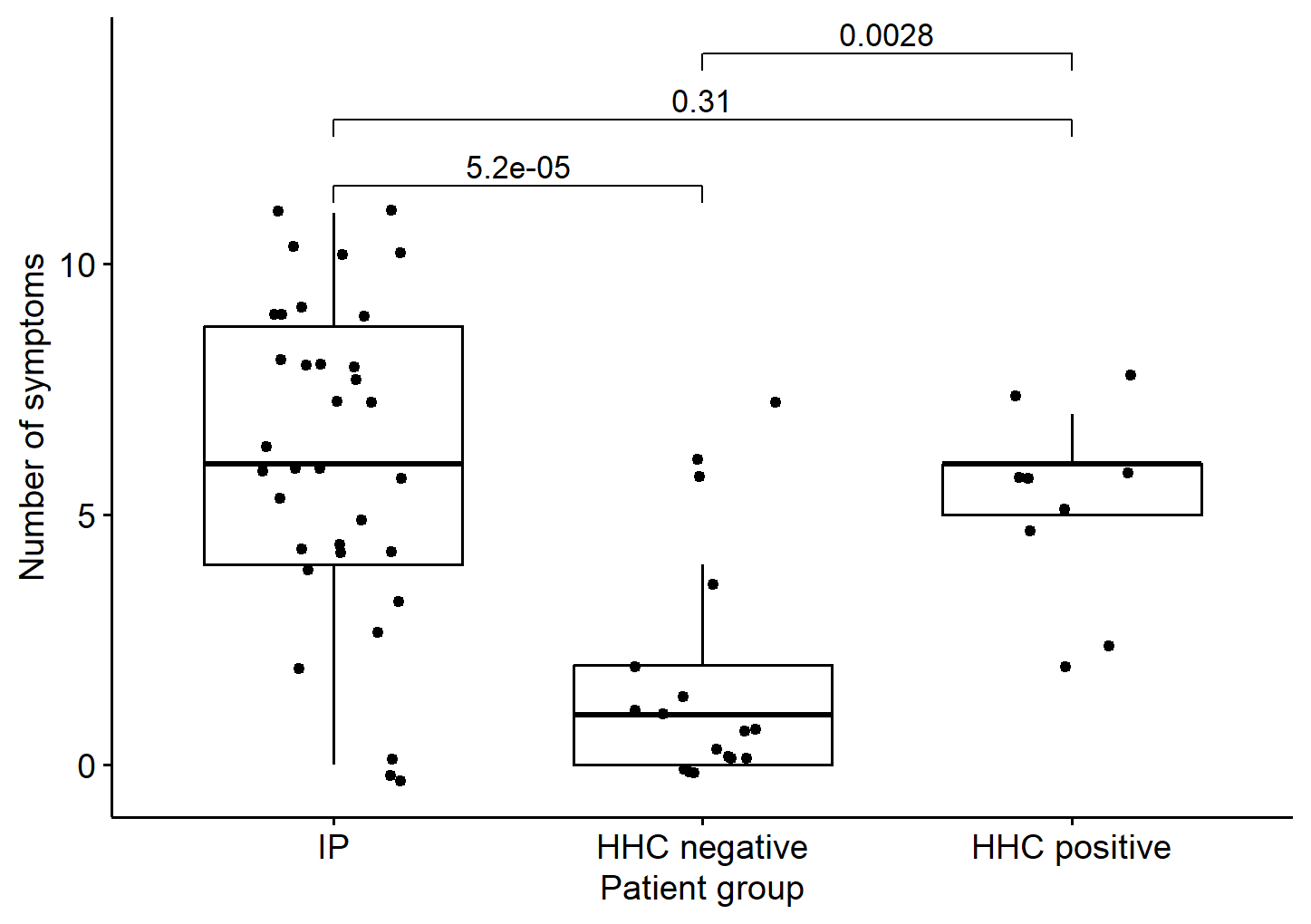


The figure shows the number of different symptoms (see Figure S3 for distinct symptom severity/occurrences) which occurred per patient over the entire study period, regardless of their severity. The mean number of symptoms differed significantly between the IP and uninfected HHC (mean number of symptoms 6.18 vs. 1.76, p < 0.001; Wilcoxon signed-rank test) and between HHC and uninfected and infected HHC (1.76 vs. 5.22, p = 0.0028). There was no significant difference in number of symptoms between IP and infected HHC (p = 0.31). IP index patient; HHC household contact.

## Figure S3. Symptom severity according to patient type.


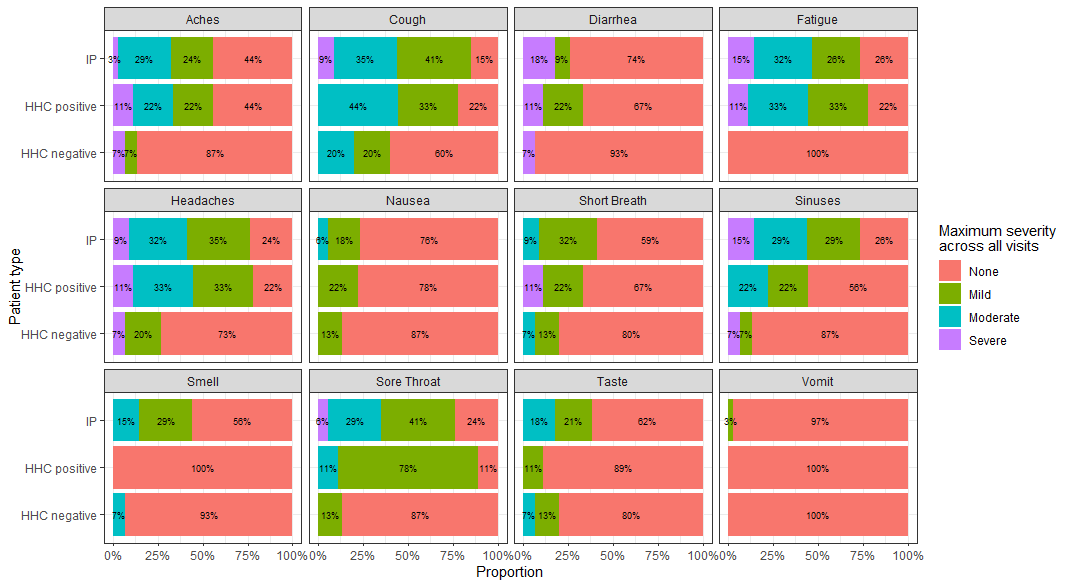


The severity of symptoms was divided into four categories: none, mild, moderate, and severe. These data are graphically represented, with no symptoms in red, mild in green, moderate in blue, and severe in purple.

## Figure S4. Kinetics of SARS-CoV-2 RNA levels.

**
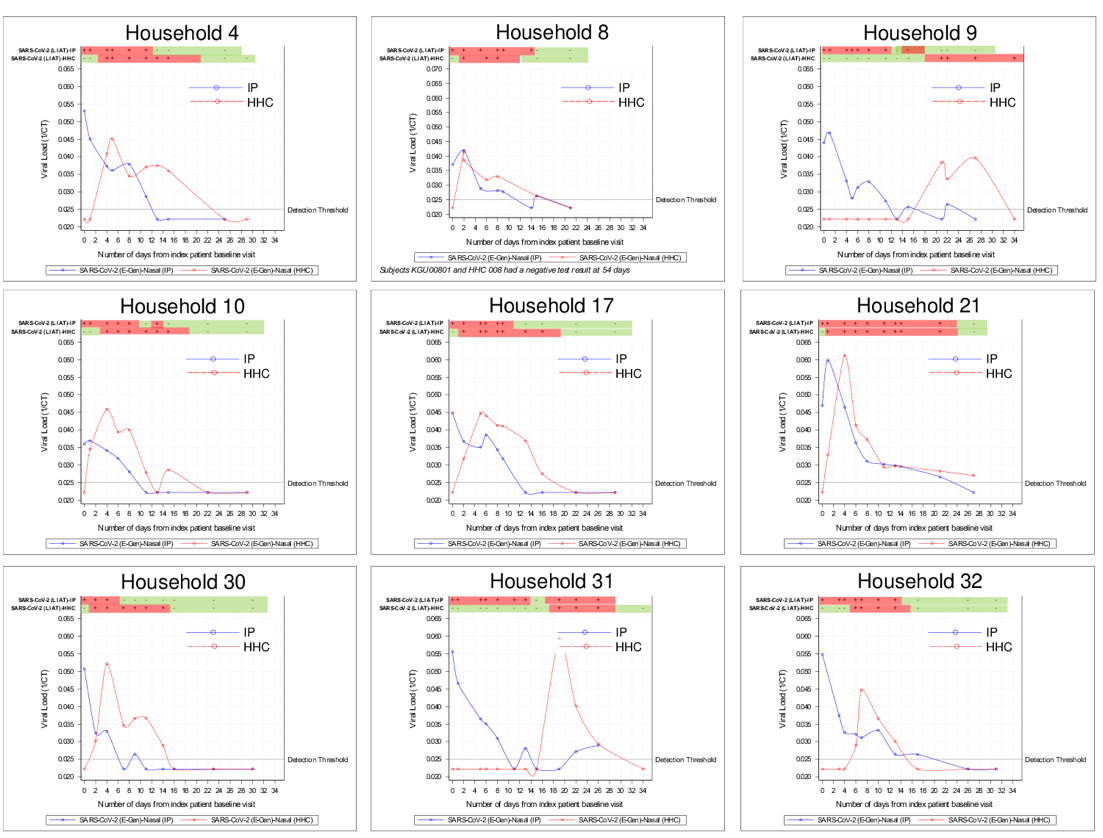
**

Each panel represents RNA levels in nasal swabs in one of the nine households in which an HHC became infected. RNA levels are shown as the inverse of the c6800 E gene Ct value (1/Ct). IP: blue lines; HHC: red lines. RNA positivity by Liat is shown at the top of each graph (red: positive; green: negative) for IP (top line) and HHC (second line).

## Figure S5. Antibody responses.

Percentage of index patients (IP, blue) or infected household contacts (HHC, red) with positive anti-N (circles) or anti-S (squares) results. Days from enrolment (IP) or first positive RNA test (HHC) are grouped as described in Methods. The number of results contributing to each data point is shown at the bottom. *Only two results contributing to the HHC percent anti-N positive at day 30; for one of these participants, antibody titers were close to the threshold for positivity between days 11 and 20 then dropped to just below the threshold on day 27 (see **Figure 4B**).

## Figure S6. Study design and testing strategy.

**(A)** Visit schedule and specimen collection. In practice, the actual timing of each visit had a window of ±1 day for the first five visits, ±2 days for visits 6 to 8, and ±3 days thereafter. Because HHC became infected after a variable amount of time from IP enrolment, for illustrative purposes we defined day 0 for infected HHC as the day or first positive RNA test by Liat or c6800. **(B)** Testing strategy.
